# Dichotomic Potency of IFNγ Licensed Allogeneic Mesenchymal Stromal Cells in Animal Models of Acute Radiation Syndrome and Graft *Versus* Host Disease

**DOI:** 10.3389/fimmu.2021.708950

**Published:** 2021-07-26

**Authors:** Raghavan Chinnadurai, Paul D. Bates, Keith A. Kunugi, Kwangok P. Nickel, Larry A. DeWerd, Christian M. Capitini, Jacques Galipeau, Randall J. Kimple

**Affiliations:** ^1^Department of Biomedical Sciences, Mercer University School of Medicine, Savannah, GA, United States; ^2^Department of Pediatrics, University of Wisconsin School of Medicine and Public Health, Madison, WI, United States; ^3^Department of Medical Physics, University of Wisconsin School of Medicine and Public Health, Madison, WI, United States; ^4^Department of Human Oncology, University of Wisconsin School of Medicine and Public Health, Madison, WI, United States; ^5^University of Wisconsin Carbone Cancer Center, University of Wisconsin School of Medicine and Public Health, Madison, WI, United States; ^6^Department of Medicine, University of Wisconsin School of Medicine and Public Health, Madison, WI, United States

**Keywords:** mesenchymal stromal/stem cells, interferon-γ, cell therapy, acute radiation injury, bone marrow transplantation, animal model

## Abstract

Mesenchymal stromal cells (MSCs) are being tested as a cell therapy in clinical trials for dozens of inflammatory disorders, with varying levels of efficacy reported. Suitable and robust preclinical animal models for testing the safety and efficacy of different types of MSC products before use in clinical trials are rare. We here introduce two highly robust animal models of immune pathology: 1) acute radiation syndrome (ARS) and 2) graft versus host disease (GvHD), in conjunction with studying the immunomodulatory effect of well-characterized Interferon gamma (IFNγ) primed bone marrow derived MSCs. The animal model of ARS is based on clinical grade dosimetry precision and bioluminescence imaging. We found that allogeneic MSCs exhibit lower persistence in naïve compared to irradiated animals, and that intraperitoneal infusion of IFNγ prelicensed allogeneic MSCs protected animals from radiation induced lethality by day 30. In direct comparison, we also investigated the effect of IFNγ prelicensed allogeneic MSCs in modulating acute GvHD in an animal model of MHC major mismatched bone marrow transplantation. Infusion of IFNγ prelicensed allogeneic MSCs failed to mitigate acute GvHD. Altogether our results demonstrate that infused IFNγ prelicensed allogeneic MSCs protect against lethality from ARS, but not GvHD, thus providing important insights on the dichotomy of IFNγ prelicensed allogenic MSCs in well characterized and robust animal models of acute tissue injury.

**Graphical Abstract:**
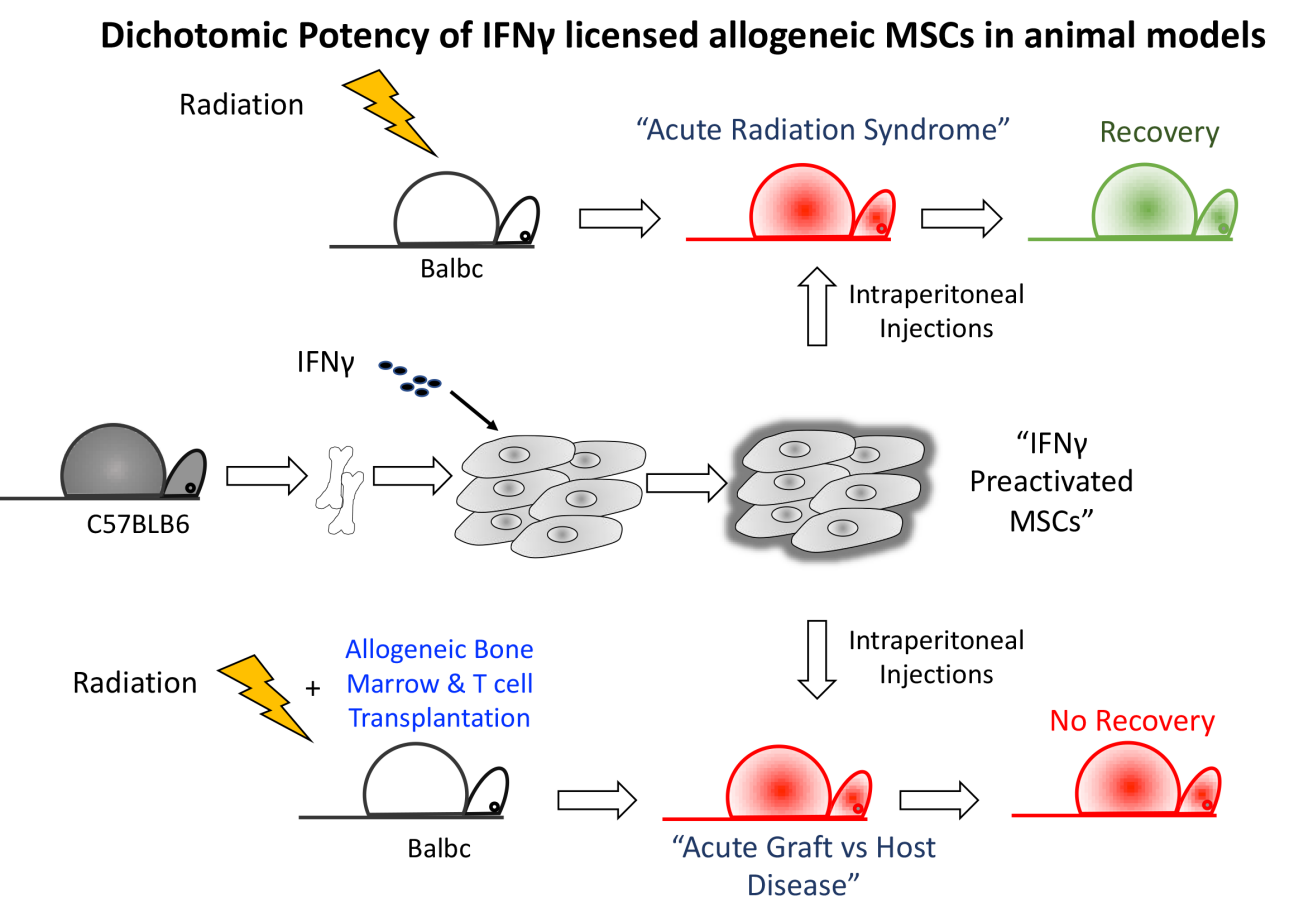


## Introduction

Mesenchymal stromal cells (MSCs) are approved in Europe for the treatment of complex perianal fistulas from Crohn’s disease and in Japan for steroid-refractory acute graft versus host disease (GvHD). MSCs are also in cell therapy in clinical trials for the treatment of a variety of inflammatory and degenerative disorders (1). Preliminary studies have showed that MSCs possess a myriad of regenerative and immunomodulatory properties and an excellent safety profile (2, 3). MSCs can be obtained from either random healthy donors (i.e. allogeneic) or from individual patients (i.e. autologous), and can be derived from multiple tissues sources including bone marrow, adipose tissue, placenta, or umbilical cord tissue (4). The use of autologous MSCs avoids potential immune-mediated tissue mismatch reactions (5, 6). However, allogeneic MSCs showed promising clinical benefits in mitigating steroid resistant Graft vs. Host Disease (GvHD) and perianal fistula in Crohn’s Disease (7–9).

While there is certainly an appeal to using autologous MSCs as the preferred source for personalized cell therapy of chronic diseases to prevent MSC rejection, the use of off-the-shelf allogeneic MSCs is more feasible for the treatment of acute inflammatory disorders which preclude the ability to harvest, isolate, and expand autologous MSCs that may have been damaged during acute tissue injury. In addition, the timing of cell therapy is significant in rescuing patients with acute illness, which is only possible with allogeneic/random donor MSC approach. Examples of this include mass casualty events causing acute radiation syndrome from exposure to Ionizing Radiation (IR) and the development of steroid resistant acute GvHD. With an allogeneic cell therapy approach, MSCs from random donors can be isolated, expanded, cryopreserved and banked as cell therapy products in advance. At the time of acute tissue injury, allogeneic MSCs can be thawed and infused into the patients immediately without the delay of lengthy cell manufacturing procedures (10). Further evidence is needed in defining which acute tissue injury conditions are amenable to the benefits of allogeneic MSCs in animal models.

Previous studies have demonstrated that IFNγ substantially improved the immunomodulatory and regenerative properties of MSCs and thus it can be an augmented cell therapy for mitigating acute tissue injury (11–15). IFNγ prelicensed allogeneic MSCs potency needs to be validated in the related animal models of acute injury. In this report, IFNγ prelicensed allogeneic MSCs were tested as a cell therapy in two models of acute tissue injury: (1) lethal ARS and (2) GvHD.

## Materials and Methods

### Murine Bone Marrow MSCs and IFNγ Licensing

All animal studies were approved by the Institutional Animal Care and Use Committee (IACUC) University of Wisconsin-Madison and Mercer University. Murine MSCs were isolated from bone marrow from the femurs and tibia of C57BL/6 animals and cultured in complete DMEM (Corning, MA, USA) (15-20% fetal bovine serum (Sigma, MO, USA) and 100U/mL penicillin/streptomycin (Corning, MA, USA). Cells were passaged/sorted to remove adherent CD45+ cells. MSC identity was confirmed as described previously (16). MSCs were used from the early passage proliferative phase. We confirm the viability of MSCs (above 90% viability) prior to infusion into the animals using trypan blue exclusion analysis. 20ng/mL IFNγ was added to MSCs and 48 hours later, cells were harvested. Exogenous IFNγ was washed and licensed MSCs were injected fresh into animals within 30-60 minutes post harvesting from the culture. IFNγ licensing of MSCs is confirmed with the evaluation of the upregulation of MHC-Class I and MHC class II expression by flow cytometry (**Figure S1**).

### ARS Model and IFNγ Licensed MSC Treatment

9 to 12 week old Balb/c animals were purchased from Jackson Laboratories (Bar Harbor, ME). Male and female animals were used to identify gender susceptibility to irradiation. Male animals were exclusively used when testing MSC therapy. One day prior to irradiation, animals were treated with Uniprim™ (Envigo, USA) and were maintained with uniprim™ diet throughout the entire experiment. On the morning of study Day 0, mice are placed in single chambers of a Plexiglas irradiation apparatus and exposed to a single uniform TBI 6MV photon irradiation from the Varian 21EX Linear Accelerator (linac) (Varian Medical Systems, Palo Alto, CA) and were irradiated with the designated dose. After 4 hours rest, animals were subjected to a second dose of irradiation. Day 1 and Day 8 post irradiation cycle, IFNγ prelicensed MSCs or PBS were given. After first week animals were supplemented with hydrogel mixed with Uniprim™ diet. Animals were monitored daily for body weight and Mouse Intervention Scoring System (MISS) score as previously described (17). Mice that reached MISS score of 12 were euthanized as described previously (17).

### Allogeneic Bone Marrow Transplantation and IFNγ Licensed MSC Treatment

C57BL/6 donor mice were euthanized by CO_2_ asphyxiation. Bone marrow (BM) cells were harvested from the tibias and fibulas using mortar and pestle and depleted of erythrocytes using ACK Lysing Buffer (Lonza, Walkersville, MD). CD3 microbeads were used on single-cell suspensions to deplete T cells using the AutoMACS Pro separation system (Miltenyi Biotec, Auburn, CA). Splenic T cells were isolated by negative selection. 4 + 4Gy irradiated Balb/c animals were injected intravenously with C57BL/6 T cell depleted BM (5X10^6^ Cells) and T cells (2X10^6^ Cells, 1X10^6^ and 0.5X10^6^) on the same day. BM alone group received only T cell depleted C57BL6 BM and not C57BL/6 splenic T cells. IFNγ licensed MSCs were infused intraperitoneally on days 2 and 8. In some situations, treatment was performed on days 1,3 and 5. Control group received only PBS. Mice were weighed individually biweekly, and the mean weight of each treatment group was calculated at each time point and was compared with the day 0 weight. Examination for moribund mice was performed by a veterinarian and veterinary technicians who were blinded to the experimental groups, and assessed the mice daily in accordance with approved institutional protocols.

### Bioluminescence Imaging

Luciferase transgenic murine MSCs (C57BL/6 background) were isolated as described previously (12). After IFNγ licensing (20ng/mL for 48 hours), exogenous IFNγ was washed and harvested cells were injected subcutaneously into naïve and irradiated animals. Each animal received subcutaneous injection of MSCs in both of its flanks. Subsequently, bioluminescence imaging was performed longitudinally on specified time points. Animals were anesthetized by isoflurane gas chamber. Subsequently, luciferin substrate was injected intraperitoneally and the animals were placed in an IVIS spectrum imager (Perkin Elmer, Waltham, MA, USA) with nose cones attached to an isoflurane gas chamber. Imaging was performed within 5-8 minutes post luciferin injection. Bioluminescence signal (Total flux) was analyzed using Living Image software (Perkin Elmer, Waltham, MA, USA) by creating a region of interest over each animal. Measurements were recorded as photons/s/cm^2^. Fold change in total flux was calculated based on the control animal that did not receive luciferase MSCs.

### Statistical Analysis

Statistical analysis was performed in GraphPad prism 8 software (GraphPad Software, San Diego, USA). An unpaired two‐sided *t*‐test was used to determine significance between the means of two groups. Kaplan Meier survival curve analysis was performed to determine the difference in percentage survival of animals between two groups. A two‐sided *p* value <.05 was considered statistically significant.

## Results

### Development of Animal Model of Radiation Injury With Clinical Grade Precision Dosimetry

**Figure 1:**
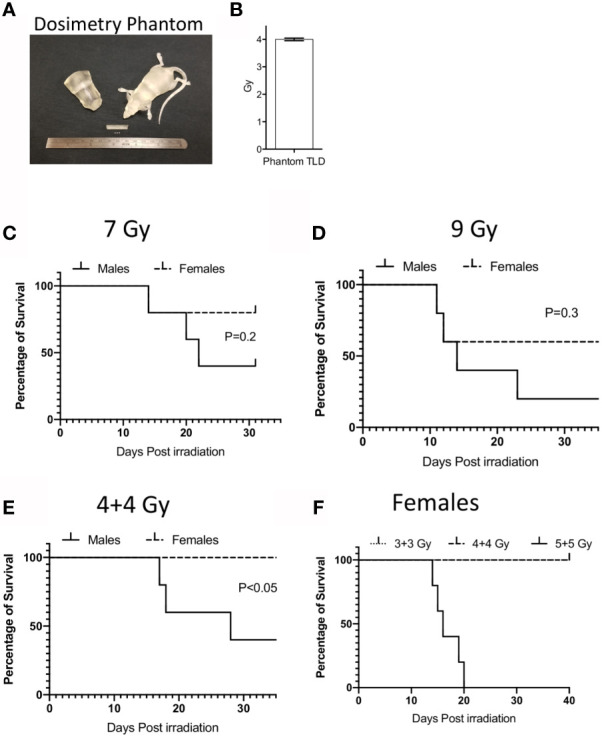
Dosimetry and IR induced lethality in male and female Balb/c mice. **(A)** Murine morphic phantom of 25g mouse/torso and 1x1x1 mm thermoluminescent dosimeters (TLD) cubes (white) are shown. TLDs can be placed within the mouse to confirm the delivered dose. **(B)** TLD measurements were shown to define the dosimetry of 4 gy IR from varian linac. Male or Female animals (n=5 per group) were subjected to single dose **(C)** 7Gy, **(D)** 9Gy or split dose irradiation with 4-hour intervals **(E)** (4 + 4 Gy). Kaplan Meier survival curves were plotted between male and female animal groups. **(F)** Dose dependent split dose irradiation was tested in females.

We utilized Balb/c mice due to their sensitivity to radiation and well-established ARS. An important issue in translating animal model of IR induced ARS is the lack of precision in radiation dose that are being used in conventional irradiators. To overcome this challenge, we have developed a model of radiation injury by integrating National Institutes of Standards and Technology (NIST)-compliant clinical grade precision dosimetry. Animals were irradiated in a Varian 21EX Linear Accelerator (Linac) with single uniform TBI 6MV photon irradiation. Linac output dosimetry rigor was verified with mouse shaped phantoms to ensure the validity of doses delivered. Each mouse irradiation cycle was verified using thermoluminescent dosimeters (TLD) 100 1 mm^3^ microcubes inside anatomically correct 3D-printed mouse phantoms (**Figure 1A**). TLDs were calibrated utilizing a ^60^Co irradiator directly traceable to NIST, as previously described (18). The uncertainty of the TLD measurements is <3.0% (**Figure 1B**). Male and female Balb/c animals were subjected to 9Gy and 7Gy total body irradiation in a single fraction. We noticed that males were more sensitive to IR induced lethality than females (**Figures 1C, D**). 9Gy and 7Gy irradiation caused more than 50% lethality at day 30 in males due to ARS. Hence, to minimize neurotoxicity we performed split doses of 4 + 4Gy irradiation with a four to five-hour interval between doses. We observed that more than 50% of males were dead by day 30 with split doses of 4 + 4Gy (**Figure 1E**). We also observed 100% lethality and severe ARS in females with the dose of 5 + 5Gy while 4 + 4Gy had no effect on females (**Figure 1F**). Since 5 + 5 Gy in females caused severe ARS, we chose to utilize males with 4 + 4Gy split dose IR as an optimal animal model to inform responsiveness to MSC therapy.

### Persistence of +/-IFNγ Licensed Allogeneic MSCs in Naïve and Irradiated Animals

**Figure 2:**
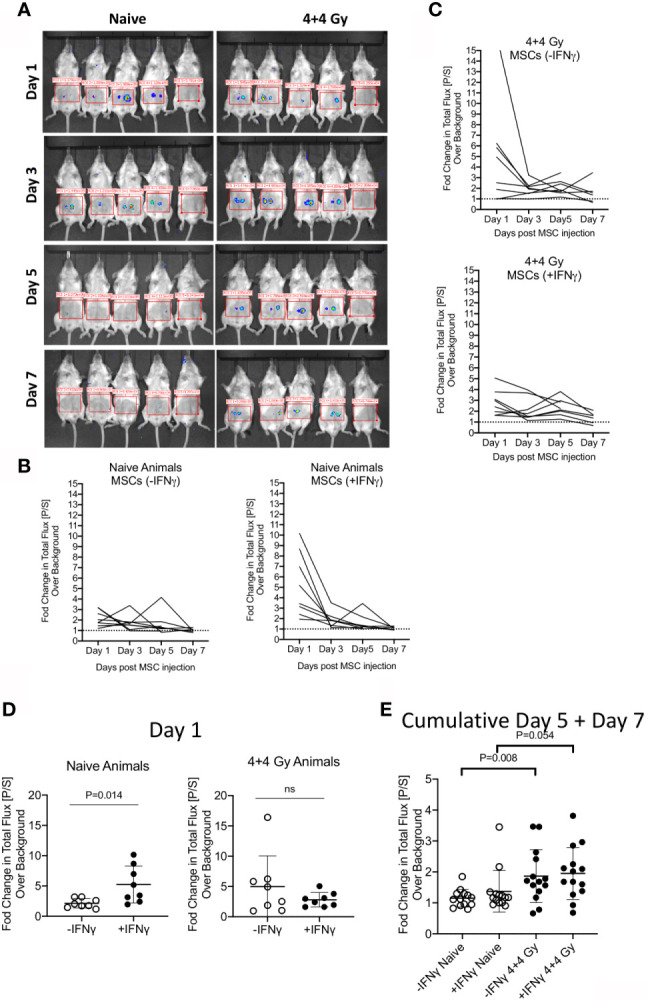
Bioluminescence imaging of +/- IFNγ licensed allogeneic MSCs on naive and irradiated animals. **(A)** Naive or 4 + 4 Gy irradiated animals were subcutaneously injected with +/- IFNγ licensed MSCs in each of the flanks. Bioluminescence imaging was performed in anesthetized animals on days 1, 3, 5 and 7. Each imaging set was performed with 5 animals which are shown as follows. -IFNγ MSCs (left two animals) +IFNγ MSCs (Middle two animals) Control animal (Rightmost). Total photon flux (photons/second) in the region of interest is measured. Fold change in bioluminescence over background is shown longitudinally in **(B)** Naive and **(C)** Irradiated animals injected with +/- IFNγ licensed MSCs. **(D)** Changes in total flux on day 1 and **(E)** days 5 + 7 are shown with +/- IFNg licensed MSCs in naive and irradiated animals. Two tailed unpaired t test was performed in GraphPad prism to obtain statistical p values. Results are shown with the cumulative from at least three independent experiments.

To define the relative fate of +/-IFNγ licensed allogeneic MSCs in naïve and irradiated animals, we performed bioluminescence imaging of infused MSCs from luciferase transgenic donors. +/- IFNγ licensed MSCs from luciferase transgenic C57BL/6 animals were injected subcutaneously (2X10^6^ cells in each flank) into male Balb/c animals that were either naïve or 4 + 4Gy irradiated 24 hours prior to injection. Animals were longitudinally imaged on days 1, 3, 5 and 7 post MSC injection (**Figure 2A**). Each set of imaging was included with control animals (without luciferase+ MSCs) and their region of interest is assigned as background luminescence. Our results demonstrated that IFNγ licensed MSCs could be readily detected at day 1 in both naïve and irradiated animals and subsequently their signal strength got diminished over time (**Figures 2A–C**). We observed statistically significant higher bioluminescence signals with IFNγ licensed MSCs compared to unlicensed MSCs in naïve animals on day 1 (**Figure 2D**). This difference was not observed at other time points (**Figure 2E**). However, we did not observe a significant difference between unlicensed and IFNγ licensed MSCs in irradiated animals (**Figure 2D**). Cumulative analysis of later time points on day 5 and day 7 has demonstrated that IFNγ licensed allogeneic MSCs were detected at the later time points in irradiated animals but not in naïve animals (**Figure 2E**). Altogether these results demonstrate that IFNγ allogeneic MSCs are rejected in naïve animals over time while in the irradiated animals they are still detected at the later time points though at lower levels compared to early time points.

### IFNγ Licensed Allogeneic MSCs Protect Animals From Lethal ARS

**Figure 3:**
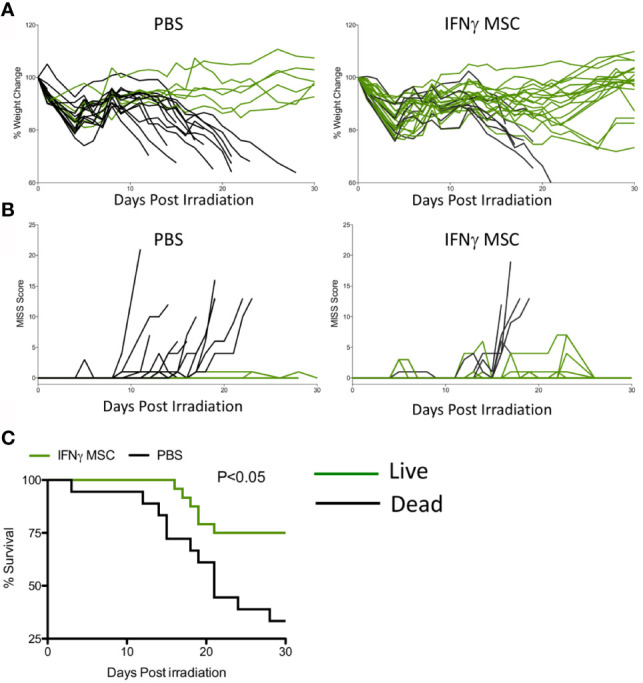
IFNγ licensed allogeneic MSCs mitigate ARS lethality. Balb/c mice were subjected to 4 + 4Gy irradiation. 24 hours later, IFNγ licensed MSCs (20ng/ml for 48 hours) were injected intraperitoneally into animals with a dose of 10^7^ cells/animal. Subsequently, a second dose of 10^7^ cells/animal was given on day 8. Controls received only PBS. **(A)** % change in body weight and **(B)** total MISS score were followed longitudinally between control (n=20 animals) and treatment groups (n=25 animals). Green lines represent mice that survived on day 30 and black lines represent dead animals. **(C)** Kaplan Meier survival curves were plotted as cumulative data from two independent groups. Cumulative of two independent experiments is shown.

We next investigated whether IFNγ licensed allogeneic MSCs could mitigate IR induced lethality. MSCs derived from C57BL/6 animals were primed with IFNγ for 48 hours before infusion. Male Balb/c animals were subjected to split doses of 4 + 4 Gy irradiation with four to four to five-hour intervals. IFNγ licensed MSCs were infused intraperitoneally with a dose of 10^7^ cells per animal on day 1 (24 hours post irradiation) and day 8. Control animals received an identical volume infusion of sterile PBS. Longitudinal analysis of animal body weight and mouse intervention scoring system suggest that IFNγ licensed allogeneic MSCs rescue animals exposed to radiation (**Figures 3A, B**). Survival curve analysis also has demonstrated that IFNγ licensed allogeneic MSCs protected animals from ARS lethality by day 30 (Survival: 6/20(Control) & 18/25(IFNγ MSC) (**Figures 3A–C**). These results demonstrate that IFNγ licensed allogeneic MSCs protect animals from lethal acute radiation syndrome.

### IFNγ Primed Allogeneic MSCs Do Not Mitigate GvHD

**Figure 4:**
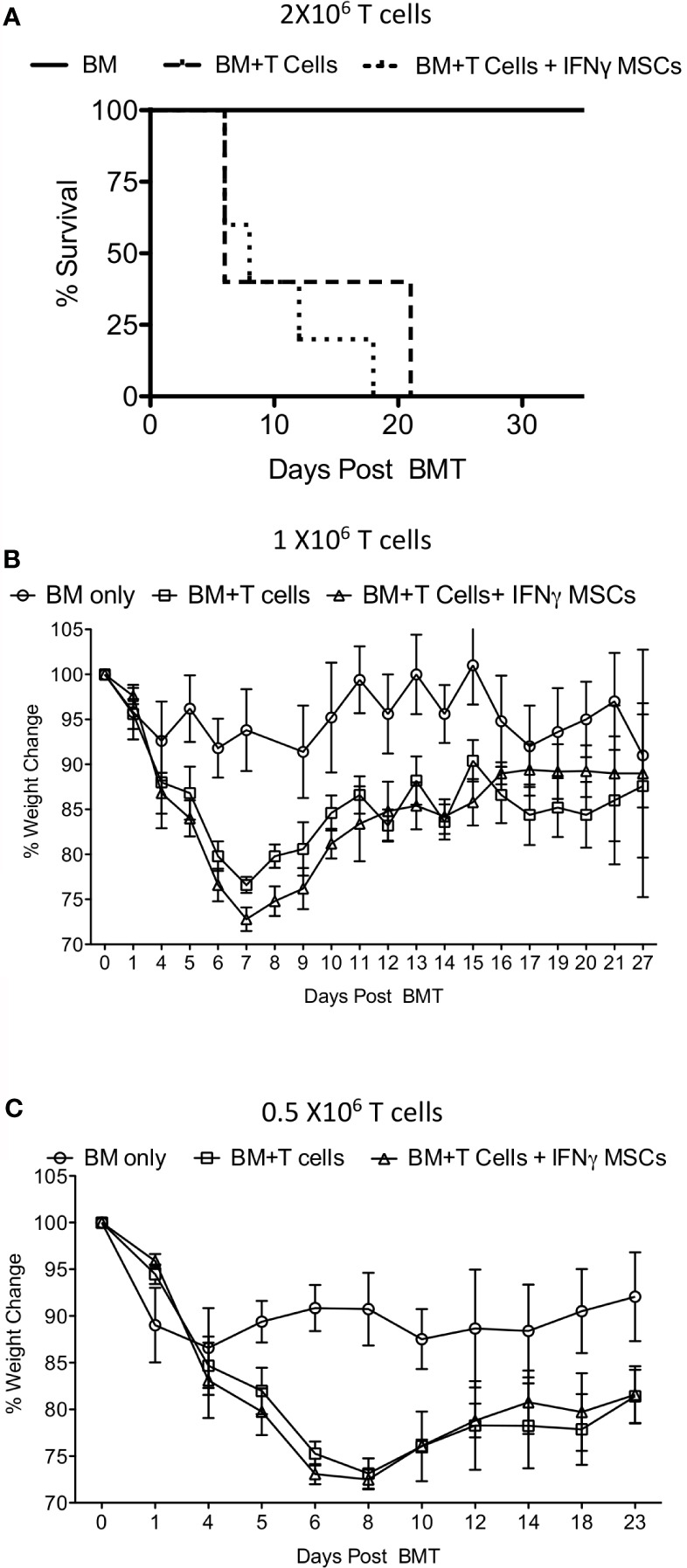
IFNγ licensed allogeneic MSCs do not mitigate GvHD from MHC mismatched allogeneic bone marrow transplantation. 4 + 4Gy irradiated Balb/c animals were transplanted with 5X10^6^ T cell depleted bone marrow and **(A)** 2 X10^6^ **(B)** 1 X10^6^ and **(C)** 0.5 X10^6^ T cells from C57BL/6 animals. Bone marrow alone group were transplanted only with 5X10^6^ T cell depleted bone marrow from C57BL/6 animals without splenic T cells. IFNγ licensed C57BL/6 MSCs (20ng/ml for 48 hours) were injected intraperitoneally into the Balb/c animals transplanted with C57BL/6 bone marrow and T cells on day 2 and day 8 post transplantation. For the experiments with 0.5X10^6^ T cells, IFNγ licensed C57BL/6 MSCs were given on days 1, 3, 5 and 8. Body weight and survival was monitored longitudinally. Each of the experiments with 2 X10^6^ 1 X10^6^ and 0.5 X10^6^ T cell and BM transplantation and MSC infusion were done independently.

Next, we investigated whether IFNγ licensed allogeneic MSCs affect GvHD induced by allogeneic bone marrow transplantation. Balb/c animals were lethally irradiated with 4 + 4Gy and subsequently transplanted with 5X10^6^ T cell depleted bone marrow and 2 X10^6^ splenic T cells from C57BL/6 donors. Animals transplanted with only 5X10^6^ T cell depleted bone marrow (no splenic T cells) were served as controls. T cell depleted bone marrow and splenic T cell transplanted animals were intraperitoneally treated with either PBS or 10^7^ IFNγ prelicensed MSCs derived from C57BL/6 on days 2 and 8 post bone marrow transplantation (BMT). IFNγ licensed MSCs did not provide any protection from lethal GvHD (**Figure 4A**). Even with reduced T cell doses to 1 X10^6^ (**Figure 4B**) and 0.5 X10^6^ (**Figure 4C**) splenic T cells per animal to cause less severe GvHD, 10^7^ IFNγ prelicensed MSCs derived from C57BL/6 animals given on days 2, 8 or days 1,3 5 post BMT did not impact GvHD (**Figures 4B, C**). Altogether, IFNγ licensed allogeneic MSCs do not impact GvHD after MHC mismatched BMT.

## Discussion

MSC based animal model studies are complicated for testing of human MSCs since immunocompetent animals may reject human cells and cytokines generated by human MSCs may not cross-react well with murine cytokine receptors. Despite these caveats, *in vivo* mouse studies have provided evidence of human MSCs mitigating inflammation and injury, but xenogenecity did confound assessment of *in vivo* MSC potency. In contrast, use of murine MSCs allows for study in immunocompetent murine models but are limited by potential lack of translatability of observations to potency and functionality of human MSCs as their mechanism of immunosuppression is distinct (16, 19). Despite these challenges, murine and human MSCs share some common pathways in mediating tissue regeneration and immunomodulation. Thus, utilization of allogeneic murine MSCs can partly inform clinical relevance.

While MSCs have shown promise for a variety of inflammatory disorders, IFNγ licensing of MSCs has the potential to substantially augment MSC engraftment, immunomodulatory properties and regenerative properties (20). Considering the significance of allogeneic/random donor MSCs in acute clinical conditions, we have tested the functionality of IFNγ prelicensed allogeneic MSCs in 2 acute animal models of lethal ARS and GvHD. In a lethal ARS model, IFNγ licensed allogeneic MSCs prolonged animal survival whereas in a lethal GvHD model, IFNγ licensed allogeneic MSCs had no impact. Previous studies have revealed the contradictory role of MSCs in mitigating GvHD in animal models (21). Allogeneic, syngeneic and xenogeneic MSCs have been shown to be ineffective in mitigating GvHD in animal models (22–28). In contrast, some studies have shown that MSCs mitigate GvHD (29–32). While variations in the strain combinations used to generate GvHD could explain the discrepancies, it is also entirely possible that MSCs do not traffic to secondary lymphoid organs and/or GvHD target organs to mitigate the associated systemic inflammation. Another explanation is that murine MSCs may not possess higher immunosuppressive properties compared to human MSCs to have a durable effect on murine donor inflammatory T cells. In addition, recent study indicate that infused MSCs undergo apoptosis and evoke host phagocytosis, which mitigate GvHD (32). Thus, the relative significance of live and apoptotic MSCs in conferring immunosuppression in modulating GvHD is yet to be confirmed in future studies. We still do not know the mechanism of action of human MSCs in mediating clinical benefit in GvHD patients. MSCs are being infused into the patients thawed from cryopreservation. It is entirely possible that thawed MSCs may undergo apoptosis and evoke an anti-GvHD response and this phenomenon yet to be proven in the future studies. Murine and human MSCs differ in their mechanism of action in executing immunosuppression (16, 19). This also could explain why human MSCs have shown benefit in clinical studies of steroid refractory GvHD while murine MSCs have no effect. One of the limitations of our study is that we used MHC mismatched BMT to generate severe GvHD while in the clinic MHC-matched, minor histocompatibility antigen mismatched BMT is typically performed. Further studies in minor histocompatibility antigen mismatched BMT may be warranted. Nevertheless, inefficacy of MSCs in the GvHD animal model does not halt the promise of utilizing human MSCs as cell therapeutics in mitigating GvHD in clinic (7, 33–36).

Previous study had shown that proinflammatory cytokine cocktail (IFNγ, IL-1β, TNFα and IFNα) activated human skin-derived precursors displayed lesser efficacy compared to unstimulated counter parts in mitigating graft vs host response in an animal model (37). In contrast, a recent study has demonstrated that cytokine cocktail (IFNγ, IL-17, IL-1β, TNFα) stimulated human cord blood tissue derived MSCs (CBti MSCs) improved the outcome of xenogeneic mouse model of GvHD (38). Although the effect of resting CBti MSCs in modulating xenogeneic GvHD is unclear, this approach is encouraging since cytokine cocktail primed CBti MSCs showed functionality even after cryopreservation and thawing (38). Utilization of cryopreserved MSCs is feasible when performing MSC therapy in multi center studies though cryopreservation and thawing affect the immunobiology of MSCs (39). Our earlier study also demonstrated that IFNγ priming of human bone marrow MSCs prior to cryopreservation protect their functionality immediately post thawing (12). In the present study we did not investigate the effect of freeze thawing on MSC’s potency. But future studies are warranted to define the effect of optimal cytokine priming technologies that allows the usage of MSCs immediately thawed from cryopreservation.

Radiation exposure is a potential public health threat for both civilians from accidental nuclear disaster and terrorist attacks, and for patients in clinical settings such as undergoing total body irradiation for BMT. The clinically available, licensed medical products for mitigation of ARS are directed toward supportive care which include (i) prophylactic antimicrobials, (ii) transfusion of platelets and erythrocytes, and (iii) G-CSF or GM-CSF. However, efficacy of these mitigators is limited since blood transfusions of platelets and erythrocytes provide only temporary correction of pancytopenia while recombinant cytokines cannot stimulate lymphocyte recovery needed to protect the host from viral infections. An international workshop on ARS at the National Institute of Allergy and Infectious Diseases (NIAID) and the *Institut de Radioprotection et de Sûreté Nucléaire* encouraged the use of bone marrow MSCs and their cytokine pre-activated derivatives as radiomitigators (40). Of importance, PLX-R18 (Pluristem Therapeutics, Inc), a placenta-derived random donor off-shelf MSC product, is being tested in an early phase clinical trial as a potential radio mitigation countermeasure. PLX-R18 is given intramuscularly to subjects who got exposure or suspected exposure of IR of ≥ 1 Gy based on Radiation Emergency Medical Management guidelines (NCT03797040). Preclinical studies have shown that xenogeneic, syngeneic or allogeneic MSCs can protect animals from lethal IR injury from ARS by ameliorating damage to the GI tract, CNS, and lungs (41–53). Our results demonstrate that IFNγ licensed allogeneic MSCs mitigate dosimetry grade ARS lethality.

Our bioluminescence imaging experiments have demonstrated that IFNγ prelicensing transiently enhances allogeneic MSC survival in the animals when given subcutaneously. However, their persistence was lost at later time points in naïve but not in irradiated animals. IFNγ induces adhesive molecules on the surface of MSCs, which include ICAM-1 (54). MSCs also express many integrin molecules and thus it is entirely possible that combination of ICAM-1 and other adhesive molecules enhance the binding of MSCs in the subcutaneous tissue (55). The faster clearance of IFNγ licensed allogeneic MSCs in naïve animals indicate that recipient immune system may be rejecting infused IFNγ licensed allogeneic MSCs. In lethally irradiated animals, this rejection process is milder compared to naïve animals. We injected MSCs 24 hours post IR and imaged after another incubation with 24 hours. IR induced inflammation and gut seepage of endotoxins may induce transient inflammation, which could explain the low-level persistence of IFNγ licensed MSCs. Despite this clearance/low persistence, IFNγ licensed allogeneic MSCs provide clinical benefit in mitigating ARS lethality, suggesting that MSC’s protective effect are mediated by their both low persistence and clearance.

Our bioluminescence imaging was performed with smaller doses (2X10^6^/each flank) of subcutaneous injections while IR protection experiments were done with larger doses (10X10^6^/animal) of intraperitoneal injections. Subcutaneous injection is limited to 0.1 ml delivery volume. 10X10^6^ MSCs are normally reconstituted into 1ml volume for intraperitoneal injections. Suspension of 10X10^6^ MSCs into 0.1ml volume for subcutaneous injections is not possible. Hence, we used smaller doses of subcutaneous delivery for bioluminescence imaging. In addition, subcutaneous delivery was performed in order to make a precise analysis on the persistence and evanescence of MSCs within the host allogeneic tissue milieu. Secondly, subcutaneous injections are precise to quantify bioluminescence imaging as the bolus is delivered into the cutis tissular microenvironment. This localized delivery has lower cellular diffusion effect in comparison to the intraperitoneal injections. Nevertheless, intraperitoneal infusion of IFNγ prelicensed MSCs in the irradiated animals also exhibit short-term persistence in irradiated animals (**Figure S2**). Altogether these data suggest that IFNγ prelicensed allogeneic MSCs mitigate IR induced lethality despite their low persistence.

We have utilized the intraperitoneal injection approach to deliver maximum number of cells (10X10^6^ cells/animal) to identify their therapeutical effect. This is only achievable with intraperitoneal but not intravenous infusion approach. Mouse cannot tolerate more than around 1X10^6^ MSCs through intravenous injection. Excessive MSCs in the venous system can cause lung embolism, which leads to the sudden death of the animals immediately post intravenous infusions. Thus, it is not possible to test the functionality of large doses of MSCs if they are given intravenously. Since the potency of mouse and human MSCs are not equivalent due to their differences in the mechanism of action, large doses of mouse MSCs are needed to be tested in the preclinical animal models. In clinical trials, MSCs are intravenously delivered up to 10X10^6^ cells per kilogram body weight in patients, which is equivalent to an intravenous infusion of 200,000 cells in a 20-gram mouse. It is unlikely to get a meaningful therapeutical effect with such a low dose of MSCs in the animal models. Despite these technical challenges, concerns have been raised regarding the intravenous administration of MSCs in some clinical situations, which can cause thromboembolism in patients due to the procoagulant tissue factor (56). Alternative routes of MSC deliveries such as intramuscular or intravascular are needed to be further investigated in the future studies. Thus, although intraperitoneal delivery approach has limitations in clinical translation, it provides a reliable mechanistic tool to test larger doses of MSCs in determining efficacy in the animal models.

ARS can cause hematopoietic, gastrointestinal, cardiovascular, or central nervous system defects (57). Similarly, GvHD is a multisymptomatic disease involving visceral organ and tissue such as gut, liver and skin. The principal difference between these two clinical indications is the involvement of allogeneic T cells in mediating inflammation. GvHD is mainly mediated by allogeneic T cell inflammation, which is absent in ARS. The contradictory clinical benefit of IFNγ licensed allogeneic MSCs in these animal models indicate that host environment plays a major role in determining the potency of MSCs. Animal model studies provide some level of insights on the potency and functionality of MSCs and their derivatives in informing human clinical cell therapy. Our results show a contrast in clinical benefit of IFNγ licensed allogeneic murine MSCs in mitigating ARS lethality versus no effect in GvHD. Caution must be exercised in translating animal model cell therapy studies into clinic.

## Data Availability Statement

The original contributions presented in the study are included in the article/**Supplementary Material**. Further inquiries can be directed to the corresponding author.

## Ethics Statement

The animal study was reviewed and approved by University of Wisconsin-Madison and Mercer University.

## Author Contributions

RC conceived and designed the studies, performed experiments, analyzed and interpreted data, and drafted the manuscript. PB and CC helped with allogeneic bone marrow transplantation experiments. KN and RK helped with radiation experiments. KK and LD helped with dosimetry experiments. JG provided critical advice on the study. RC, CC, and RK edited the manuscript. All authors contributed to the article and approved the submitted version.

## Funding

This study was directly supported by the WES Leukemia Research Foundation (RC). This research was also supported by Mercer University School of Medicine’s research funds (RC) and generous support from the Landings Women’s Golf Association (Savannah, GA). This work was also supported by National Institutes of Health, National Institute of Diabetes and Digestive and Kidney Diseases award R01DK109508 (JG). This study was also supported by a St. Baldrick’s-Stand Up To Cancer Pediatric Dream Team Translational Research Grant SU2C-AACR-DT-27-17, NIH/NCATS UL1TR000427 to the UW ICTR and NIH/NCI P30 CA014520 to the UWCCC, and NIH/NCI R01 CA215461 (CC). Stand Up To Cancer is a division of the Entertainment Industry Foundation. Research grants are administered by the American Association for Cancer Research, the scientific partner of SU2C. The contents of this article do not necessarily reflect the views or policies of the Department of Health and Human Services, nor does mention of trade names, commercial products, or organizations imply endorsement by the US Government. None of these funding sources had any input in the study design, analysis, manuscript preparation or decision to submit for publication.

## Conflict of Interest

CC receives honorarium from Nektar Therapeutics.

The remaining authors declare that the research was conducted in the absence of any commercial or financial relationships that could be construed as a potential conflict of interest.

## Publisher’s Note

All claims expressed in this article are solely those of the authors and do not necessarily represent those of their affiliated organizations, or those of the publisher, the editors and the reviewers. Any product that may be evaluated in this article, or claim that may be made by its manufacturer, is not guaranteed or endorsed by the publisher.
