# Supplementary material for: Dichotomic Potency of IFNγ Licensed Allogeneic Mesenchymal Stromal Cells in Animal Models of Acute Radiation Syndrome and Graft Versus Host Disease
Source: Front Immunol. 2021 Jul 26;12:708950. doi: 10.3389/fimmu.2021.708950 (PMC8352793; doi:10.3389/fimmu.2021.708950)
Supplement: Supplementary file 1 [file Image_1.pdf]

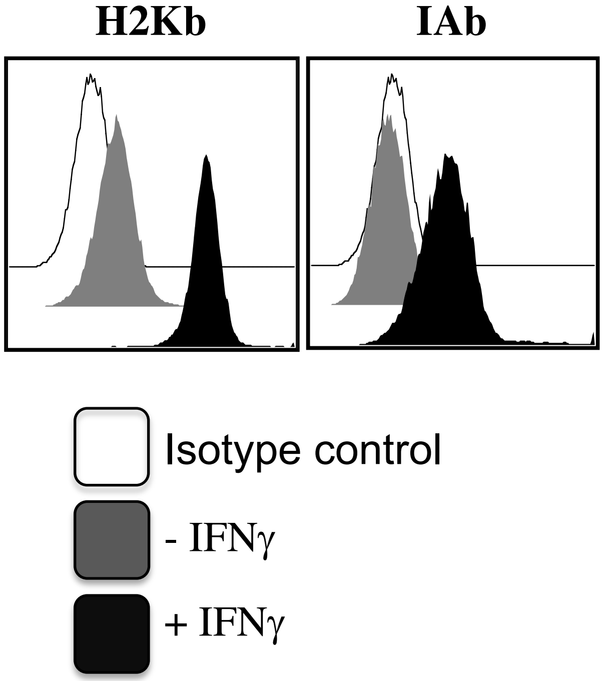

Figure S1. MHC class I and MHC Class II expression on IFNγ licensed MSCs. C57BL/6 MSCs were stimulated with IFNγ for 48 hours and subsequently surface expression of MHC class I(H2K) and MHC class II (Iab) was measured in flow cytometry.
