# Supplementary material for: Dichotomic Potency of IFNγ Licensed Allogeneic Mesenchymal Stromal Cells in Animal Models of Acute Radiation Syndrome and Graft Versus Host Disease
Source: Front Immunol. 2021 Jul 26;12:708950. doi: 10.3389/fimmu.2021.708950 (PMC8352793; doi:10.3389/fimmu.2021.708950)
Supplement: Supplementary file 2 [file Image_2.pdf]

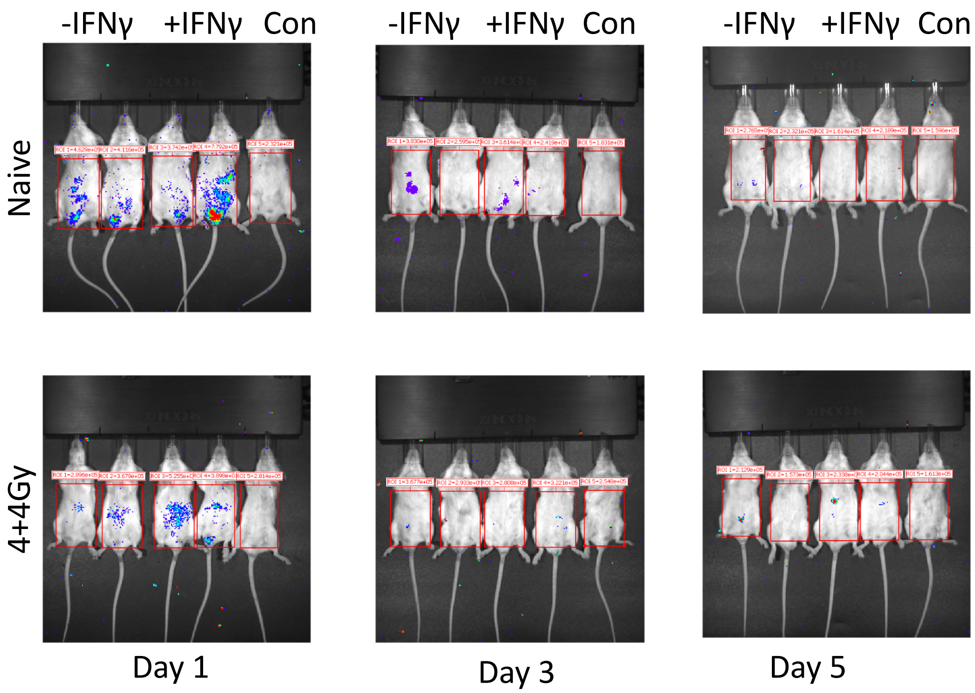

Figure S2. Bioluminescence imaging of intraperitoneally delivered IFNγ licensed allogeneic MSCs. Balb/c animals were subjected to 4+4Gy irradiation. 24 hours later, IFNγ (20ng/ml for 48 hours) licensed luciferase+ C57BL/6 MSCs were injected intraperitoneally with a dose of 10^7 cells/animal. Bioluminescence imaging was performed on days 1, 3 and 5 post injection.

Figure S2
